# Nora virus proliferates in dividing intestinal stem cells and sensitizes flies to intestinal infection and oxidative stress

**DOI:** 10.1101/2025.01.30.635658

**Published:** 2025-02-04

**Authors:** Adrien Franchet, Samantha Haller, Miriam Yamba, Vincent Barbier, Angelica Thomaz-Vieira, Vincent Leclerc, Stefanie Becker, Kwang-Zin Lee, Igor Orlov, Danièle Spehner, Laurent Daeffler, Dominique Ferrandon

**Affiliations:** 1 UPR 9022 CNRS, IBMC, University of Strasbourg, France.; 2 UMR 7104 CNRS, U964 INSERM, IGBMC, University of Strasbourg, France.; 3 Institute for Parasitology and Research Center for Emerging Infections and Zoonoses, University of Veterinary Medicine Hannover, Hannover, Germany.; 4 Present address: The Francis Crick Institute, London, UK.; 5 Present address: Fraunhofer Institute for Molecular Biology and Applied Ecology (IME), Ohlebergsweg 12, Giessen, Germany.; 6 Present address: UMR 7178 CNRS, Institut Pluridisciplinaire Hubert Curien, Strasbourg, France.; 7 Present address: Institute of Translational Medicine and Liver Disease, Inserm U1110, Strasbourg, France.

## Abstract

The digestive tract represents the most complex interface of an organism with its biotope. Food may be contaminated by pathogens and toxicants while an abundant and complex microbiota strives in the gut lumen. The organism must defend itself against potentially noxious biotic or abiotic stresses while preserving its microbiota, provided it plays a beneficial role. The presence of intestinal viruses adds another layer of complexity. Starting from a differential sensitivity of two lines from the same *Drosophila* wild-type strain to ingested *Pseudomonas aeruginosa*, we report here that the presence of Nora virus in the gut epithelium promotes the sensitivity to this bacterial pathogen as well as to an ingested oxidizing xenobiotic. The genotype, age, nature of the ingested food and to a limited extent the microbiota are relevant parameters that influence the effects of Nora virus on host fitness. Mechanistically, we detect the initial presence of viral proteins essentially in progenitor cells. Upon stress such as infection, exposure to xenobiotics, aging or feeding on a rich-food diet, the virus is then detected in enterocytes, which correlates with a disruption of the intestinal barrier function in aged flies. Finally, we show that the virus proliferates only when ISCs are induced to divide and that blocking either enterocyte apoptosis or JAK/STAT-driven ISC division leads to a drastically reduced Nora virus titer.

In conclusion, it is important to check that experimental strains are devoid of intestinal viruses when monitoring survival/life span of fly lines or when investigating the homeostasis of the intestinal epithelium as these viruses can constitute significant confounding factors.

## Introduction

The digestive tract represents the most complex interface of the host with its environment as it involves a large surface, the presence of an important microbiota, the ingestion of food potentially contaminated by pathogens or toxicants, and the need for absorption of digested nutrients and solutes. Multiple concurrent infections or abiotic stresses are likely to be common, especially when the host feeds on decaying organic matter, as is the case for *Drosophila melanogaster*. Co-infection models with bacterial and viral pathogens in the gut are starting to be experimentally investigated and have revealed an enhanced susceptibility to combined infections that can, be caused either by affecting pathogen transmission or by an inability to withstand tissue damage (Lian et al., 2022, and references therein).

Some twenty distinct viruses have been identified in wild Drosophila and around 30% of wild individuals are infected by at least one virus (Webster et al., 2015). A few of them are studied in laboratory for the mechanisms of interaction with the host: *Drosophila melanogaster* Sigma Virus (DMelSV), *Drosophila* A virus (DAV), *Drosophila* C virus (DCV), Drosophila X virus (DXV), and Nora virus (Schneider and Imler, 2021; Webster et al., 2015). Nora is an enteric virus (Habayeb et al., 2006). Unlike other insect picorna-like viruses, its genome encodes four open reading frames corresponding to: a suppressor of RNA interference, VP1 (van Mierlo et al., 2012), replicative proteins coded by ORF2, the poorly characterized product of ORF3, and capsid proteins derived from ORF4 (Ekstrom et al., 2011). This virus infects common laboratory stocks where it appears to cause a persistent infection. It is transmitted horizontally and vertically via a fecal-oral route (Habayeb et al., 2009a; Habayeb et al., 2006). The Nora virus likely proliferates in the digestive tract, as large quantities of the virus are continuously released in the feces of infected flies. The virus does not appear to have major effects on host fitness, even though some damages to the intestinal epithelium have been reported (Habayeb et al., 2009a). However, the microbiota of flies infected by Nora may change in quantity and bacterial diversity (Schissel et al., 2021). We then wondered whether a persistent Nora virus infection could influence a secondary pathogenic bacterial infection.

The *Drosophila* intestinal epithelium is simple, formed mostly by a monolayer of columnar polyploid enterocytes (EC) and also comprises enteroendocrine cells and intestinal stem cells (ISCs) and enterocytes progenitor called enteroblasts (Jiang and Edgar, 2011; Lemaitre and Miguel-Aliaga, 2013). The microbiota is made up of few species, at most twenty, and is usually dominated by two-three prevalent species such as *Acetobacter pomorum*, *Lactobacillus plantarum*, or *Enterococcus faecalis,* but its composition changes with ageing (Broderick and Lemaitre, 2012).

Immune defenses in the midgut include a chemical armentamarium, notably reactive oxygen species (ROS) generated by the NADPH oxidase, and likely not Dual oxidase, and antimicrobial peptides such as Diptericin (Ayyaz and Jasper, 2013; Buchon et al., 2013; Ferrandon, 2013; Kim and Lee, 2014; Liu et al., 2024). Resilience, a complementary dimension of mucosal host defense also known as disease tolerance (Medzhitov et al., 2012), is the ability of the intestinal epithelium to maintain its homeostasis, for instance by a mechanism of cytoplasmic purge (Lee et al., 2016) or the increased proliferation of ISCs that ultimately regenerate enterocytes damaged either directly by pathogens or indirectly by the host’s own immune response (Ferrandon, 2013, and references therein). ISC proliferation is regulated by the production of the growth factor Unpaired 3 (Upd3) that activates the JAnus Kinase/Signal Transducers and Activators of Transcription (JAK/STAT) pathway in ISCs (Buchon et al., 2009; Herrera and Bach, 2019). Several models of gut infection have been developed in *Drosophila* (Bonfini et al., 2016) and can be used to study coinfection with Nora virus. We have focused on *Serratia marcescens* and *Pseudomonas aeruginosa* intestinal infections (Chen et al., 2024; Cronin et al., 2009; Haller et al., 2018; Lee et al., 2016; Limmer et al., 2011a; Nehme et al., 2007; Sina Rahme et al., 2022; Socha et al., 2023) and had not noticed major damages of *P. aeruginosa* oral infections on the integrity of the intestinal epithelium in contrast to another study (Apidianakis et al., 2009). *P. aeruginosa* bacteria are able to cross the intestinal barrier and to silently colonize inner tissues (Chen et al., 2024). They are initially found circulating in the hemolymph at low concentration. However, after a few days of continuous feeding on bacterial solution, the bacteria start proliferating in the hemolymph in a quorum-sensing-dependent manner and ultimately kill the host through bacteremia (Haller et al., 2018; Limmer et al., 2011a).

Here, we demonstrate that Nora virus is a co-factor that synergizes with ingested bacteria or toxicants in *Drosophila* intestinal infection models, thus leading to an earlier demise of infected flies. We also report major effects of Nora infection on the lifespan of the flies under different feeding conditions. Mechanistically, we find that Nora virus infects originally ISCs and proliferates intensely upon ISCs divisions, ultimately leading to the contamination of enterocytes and gut barrier function disruption. Blocking the compensatory ISC proliferation by either inhibiting apoptosis in enterocytes or interfering with JAK-STAT signaling in ISCs protects flies from Nora pathogenesis through a drastic decrease of its titer.

## Results

### Nora virus-infected stocks are shorter-lived and more susceptible to some infections

**Figure 1:**
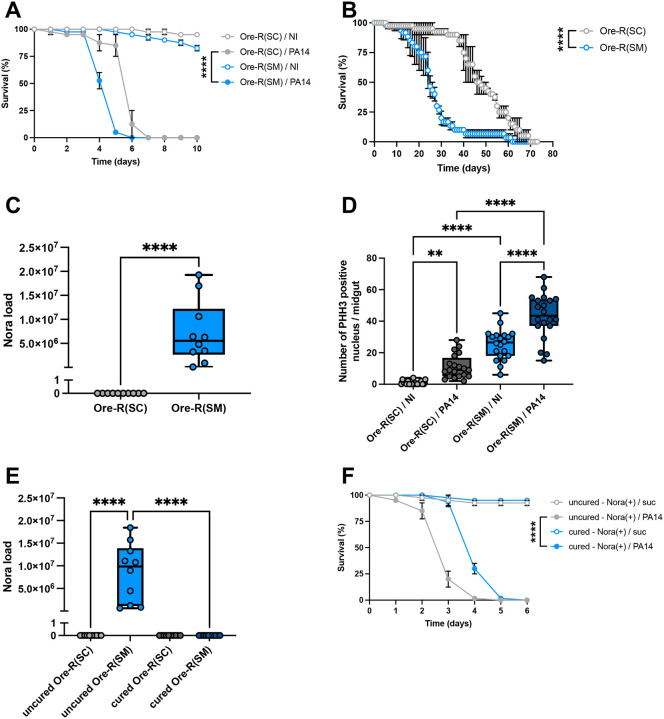
Infested Nora flies have a weaker fitness and are more sensitive to various stress conditions Flies infested with Nora virus are noted as Ore-R(SM) and non-infested flies as Ore-R(SC). (A) Ore-R(SM) (Nora(+)) were more susceptible to an intestinal infection with PA14 in survival experiments at 25°C. Infected flies: PA14; noninfected control flies: NI. (B) Nora infested flies presented a reduced lifespan compared to uninfested flies. (C) Nora titer of Ore-R(SM) and Ore-R(SC) stocks measured by qRT-PCR. (D) A significant difference of the number PHH3 positive nuclei in *Drosophila* midgut at 2 days after an intestinal infection with PA14 (PA14) or noninfected controls (NI) was observed between Ore-R(SM) (Nora(+)) and Ore-R(SC) (Nora(−)) flies. (E) Nora virus titer of the original and cured stocks as measured by qRT-PCR. (F) Flies used for these experiments were all originally OreR(SM) *Drosophila* infested with the Nora virus. Some flies have been cured from Nora virus (Nora(−)) by bleaching the eggs and kept separately from the Nora virus infested flies (Nora(+)). All data represent the mean of biological triplicates ± SEM of one representative experiment out of three. LogRank test was performed for survival test. Mann-Whitney nonparametric t-test was performed for Nora load. One-Way ANOVA with multiple comparison was perform for PHH3 quantification. *p<0.05, **p<0.01, ***p<0.001, ****p < 0.0001.

We noted that two Oregon-R (Ore-R) wild-type stocks kept by different investigators in the laboratory, hereafter referred to as (SM) and (SC), displayed a remarkably distinct survival pattern in one out of two models of intestinal infections (Limmer et al., 2011a; Nehme et al., 2007) in which flies respectively fed either on *Pseudomonas aeruginosa* PA14 (Fig. 1A) or *Serratia marcescens* Db11 (Fig. S1A). Namely, the Ore-R (SM) was more sensitive to PA14 and there was a slight trend toward higher susceptibility to Db11. We also noted that Ore-R (SM) displayed a shorter lifespan under non-infected conditions either on our standard food or on sucrose solution (Fig. 1B, Fig. S1B, and see below). We wondered whether the difference might be due to an infection and checked the stocks for the presence of common microbes known to affect *Drosophila* stocks (Haller et al., 2014; Lestradet et al., 2014). The only notable difference we identified between the two stocks was the presence of the Nora enteric virus in the infection-sensitive Ore-R(SM) (Fig. 1C).

We therefore examined the guts of flies from the two Ore-R stocks and did not detect any major morphological differences. However, when we measured basal ISC proliferation by counting phospho-histone H3-positive (PHH3) cells in the intestine, we found an enhanced rate of ISC proliferation in the Nora-positive Ore-R(SM) stock (Fig. 1D). When we challenged the Nora-negative Ore-R(SC) stock with *P. aeruginosa*, we found a small but nevertheless significant increase in ISC proliferation. However, this *P. aeruginosa*-induced increase was three-times larger in the Nora-positive Ore-R(SM) stock (Fig. 1D).

Overall, these observations suggest that flies infected with Nora have a lower fitness and are more susceptible to infections than non-infected flies.

### The Nora virus causes the enhanced susceptibility to *P. aeruginosa* intestinal infections

As expected, bleaching the eggs laid by the Nora-infected Ore-R(SM) stock appeared to eradicate the virus ((Habayeb et al., 2009b), Fig. 1E and Fig. S2A). This treatment did not have a noticeable adverse impact as bleached stocks displayed a normal survival for at least ten days on sucrose (Fig. S1E–I). Furthermore, cured Ore-R(SM) flies fed with *P. aeruginosa* were less susceptible to this intestinal challenge than the uncured stock (Fig. 1F).

We placed our cured flies in a vial that had hosted infected males for several days for fecal-oral transmission (Habayeb et al., 2009a) and found that they became again Nora-positive over several generations (Fig. S2A). The reinfected stock was more sensitive to the ingestion of *P. aeruginosa* than the cured stock (Fig. S2B). This correlated with an enhanced ISC proliferation in the Nora virus reinfected flies, whether challenged with *P. aeruginosa* or not (Fig. S2C).

**Figure 2:**
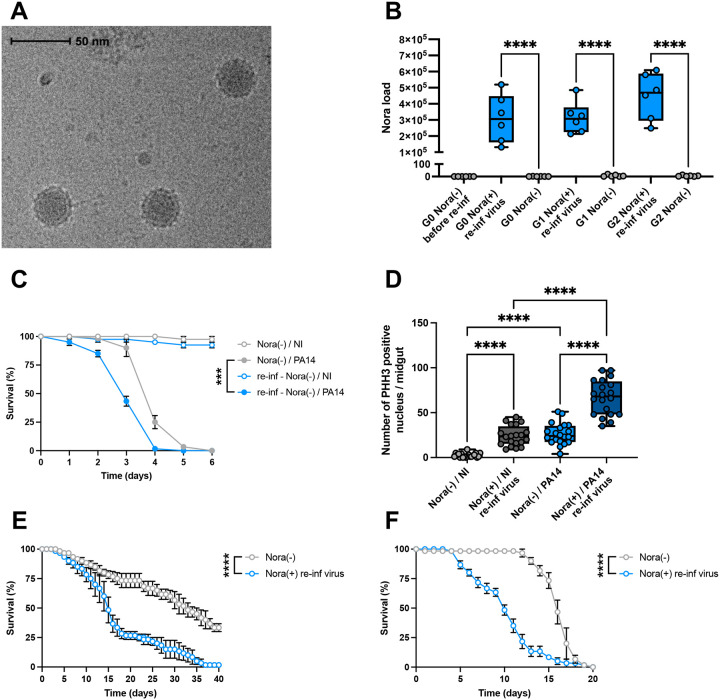
Nora virus causes an enhanced susceptibility to PA14 intestinal infections Flies used for these experiments were from Ore-R(SM) stocks cured from Nora infestation by egg bleaching. Some of these flies were re-infected with a pure suspension of Nora virus (Nora(+) re-inf virus) or not (Nora(−)). (PA14) were the flies infected with PA14 and (NI) the uninfected control flies. (A) Electronic microscopic picture of the pure Nora virus preparation. Black arrows indicate Nora virus particles. (B) A significant difference in the Nora virus titer measured by qRT-PCR was observed between Nora(+) and Nora(−) flies in the first (G1) and second (G2) generation after re-infection with virus. The observed difference between the initially re-infected G0 generation flies (G0 Nora(+) re-inf virus) or not (G0 Nora(−)) was not significant. (C) Nora(+) flies were more susceptible than Nora(−) flies to an intestinal infection with PA14 at 25°C. (D) A difference in the number of PHH3 positive nuclei in midguts was observed between Nora(+) and Nora(−) flies whether the flies had ingested PA14 for two days or not. A significant increase of PHH3 positive nuclei was detected after a PA14 intestinal infection in both Nora(+) and Nora(−) flies. (E) Nora(+) flies displayed a shorter lifespan than Nora(−) flies. (F) A marked increase of the death rate on a sucrose diet was observed in Nora(+) flies compared to Nora(−) flies. All data represent the mean of biological triplicates ± SEM of one representative experiment out of three. LogRank test was performed for survival test. Mann-Whitney nonparametric t-test was performed for Nora load. One-Way ANOVA with multiple comparison was perform for PHH3 quantification. *p<0.05, **p<0.01, ***p<0.001, ****p < 0.0001.

The drawback of the fecal transmission route is that other enteric pathogens may be transferred along with the Nora virus. To exclude this possibility, we used gradient centrifugation to purify and concentrate Nora virus from an infected fly extract. This preparation was homogenous with particles of the expected size when observed by cryo-electron microscopy (Fig. 2A). Using RT-PCR, we confirmed the identity of the virus and ruled out the presence of other known contaminating *Drosophila* viruses (DAV, DCV, DBV, DTV, DXV, CrPV, FHV, *etc*…; see Table 1 Primer Sequence). Cured flies were fed on this pure viral preparation for 24 hours. This was sufficient to stably reinfect the stock over several generations (Fig. 2B). As with the fecal contamination route, we observed that the flies reinfected with the pure viral preparation were more sensitive to the ingestion of *P. aeruginosa* (Fig. 2C) and displayed an enhanced rate of ISC proliferation in the gut (Fig. 2D). In terms of lifespan on standard food or on sucrose solution, the re-infected stocks displayed a largely decreased fitness (Fig. 2E–F), in contrast to the findings of (Habayeb et al., 2009a; Nigg et al., 2024) who reported only a mild effect upon the injection of the Nora virus. Altogether, these results demonstrate that Nora virus is responsible for the enhanced sensitivity to *P. aeruginosa* intestinal infection.

We suspected that a polymorphism in the gene *pastrel* might be the cause of the different susceptibility to Nora virus between Ore-R(SM) and Ore-R(SC) (Magwire et al., 2012). To test this hypothesis, we determined by RT-PCR the presence of a *pastrel* sensitive or resistant alleles (*pst*^*S*^, *pst*^*R*^) in several stocks commonly used in the laboratory. Strikingly, the sensitive allele was found in homozygous condition only in the Ore-R (SM) stock, while it was heterozygous in our *w* (A5001) stock (Fig. S1D). Interestingly, the *w* (A5001) (Thibault et al., 2004) and the DD1 *cn bw* (Jung et al., 2001) stocks were found to be also harboring the Nora virus, the latter one being *pst*^*R*^*/ pst*^*R*^ (Fig. S1C). The w(A5001) and DD1 *cn bw* cured stocks as well as the Canton-S stocks were all more susceptible to *P. aeruginosa* ingestion after having been converted to a Nora-positive status by the prior ingestion of the purified virus preparation (Fig. S1E–G). Since even stocks harboring two copies of the resistant *pastrel* allele became more sensitive to ingested *P. aeruginosa* (Fig. S1D, S1F, G), we can rule out that distinct *pastrel* alleles were also the cause of the enhanced sensitivity to bacterial intestinal infections. Unexpectedly, the ORE-R (SC) strain appeared to be resistant to the ingestion of Nora since it did not display an enhanced sensitivity to oral *P. aeruginosa* infection (Fig.S1I). Thus, this line may harbor in addition to the homozygous *pst*^*R*^ allele an unidentified locus that restricts the Nora virus.

In the following, we will use the Ore-R(SM) cured stock as a Nora-negative control. We will further characterize the impact of Nora virus on infected flies using the cured Ore-R(SM) stock reinfected with the pure Nora preparation, which will be referred to as Nora-positive flies.

### Nora virus is reducing host longevity largely independently of the microbiota

**Figure 3:**
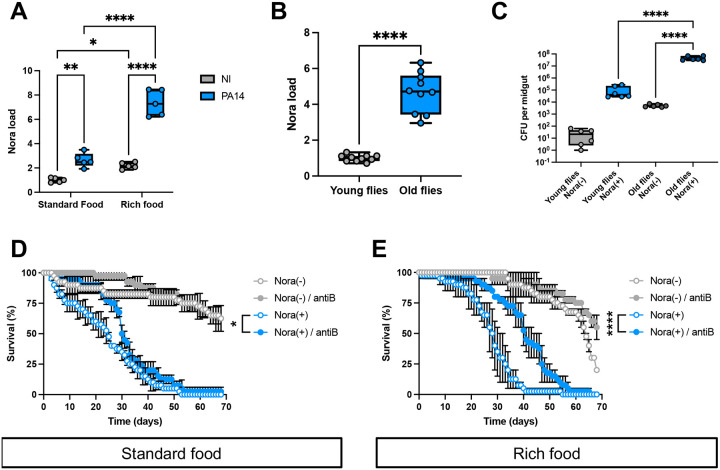
Aging, nutrient, and microbiota increase susceptibility of Nora infested flies to PA14 infections (A) Quantification of Nora virus by RT-qPCR at 3d post-infection from flies infested or not from standard food or rich food (= standard medium plus 5x extra yeast) with PA14 coinfection. (B) A significant difference in the Nora virus titer measured by qRT-PCR was observed between Nora(+) young (3d old) versus old (30d old) flies. (C) A strong increase of four orders of magnitude (Log scale) of the microbiotal titer was observed between young Nora(+) and Nora(−) flies kept for eight days on a sucrose diet. A significant increase was detected in old flies kept on standard fly food. (D) Nora infested flies presented a reduced lifespan compared to uninfested flies on standard food with or without the addition of antibiotics (antiB). (E) Nora infested flies presented a reduced lifespan compared to uninfested flies on rich food (= standard medium plus 5x extra yeast) with or without the addition of antibiotics (antiB). All data represent the mean of biological triplicates ± SEM of one representative experiment out of three. LogRank test was performed for survival test. Mann-Whitney nonparametric t-test was performed for Nora load. One-Way ANOVA with multiple comparison was perform for PHH3 and CFU quantification. *p<0.05, **p<0.01, ***p<0.001, ****p < 0.0001.

We confirmed that Nora re-infected flies succumbed much earlier than Nora-negative control flies when orally challenged with *P. aeruginosa* in a sucrose solution after five days of prior feeding on our standard food or a rich food corresponding to our standard food containing five times more yeast (Fig. S3A–B). Strikingly, we observed that an oral infection with PA14 increased the Nora burden (thrice on standard food and four-times on rich food). Actually, the Nora virus titer and the *P. aeruginosa* load was higher for flies fed on rich food (Fig. 3A, Fig. S3C). Of note, flies fed on rich food succumbed earlier to the challenge, independently of their Nora status (compare S3B to S3A). We noted a correlation between the Nora titer in the absence or presence of PA14 and the measured proliferation of ISCs, which held also on rich food (compare Fig. 3A and Fig. S3D).

As aging flies exhibit a dysbiosis that correlates with an impaired homeostasis of the intestinal epithelium (Biteau et al., 2008), we also compared the Nora burden in young (3–5 day-old) and old (30–35 day-old) flies and found an about four-fold increase (Fig. 3B), which correlated with an enhanced rate of ISC proliferation (Fig. S3E), in keeping with a previously published study (Hanson and Lemaitre, 2023). While checking for an increase of the microbiota load in old flies (Iatsenko et al., 2018), we noted that the microbiota titer was much higher in Nora-positive than in Nora-negative flies (Fig. 3C). This observation also held when flies were kept feeding only on a sucrose solution (Fig. S3F). To determine whether the microbiota influenced the fitness of Nora-negative or -positive flies, we first monitored their survival when kept on sucrose solution. An antibiotic mix treatment substantially decreased the lifespan of Nora-negative flies, possibly reflecting a positive impact of the microbiota on this amino-acid depleted food solution. In contrast, antibiotics treatment had no effect on Nora-positive flies (Fig. S3G). Interestingly, the proliferation of ISCs was increased in antibiotics-treated Nora-positive flies (Fig. S3H), which inversely correlates with the life span of those flies. We next monitored the fitness of Nora-negative or -positive flies on our standard or on rich food. The antibiotic treatment had a limited effect on Nora-negative flies with a somewhat enhanced fitness (Fig. 3D–E). In contrast, it significantly extended the lifespan of Nora-positive flies on either standard or rich food (note that the detrimental effect of the Nora virus is attenuated on rich food, at least for two-thirds of their lives). However, the antibiotics treatment failed to protect the flies to the level of Nora-negative flies. We conclude that under these different feeding conditions, the microbiota contributes at best only partially to the decreased fitness of Nora-positive flies.

### Nora virus contamination impairs the barrier function of the intestinal epithelium

**Figure 4:**
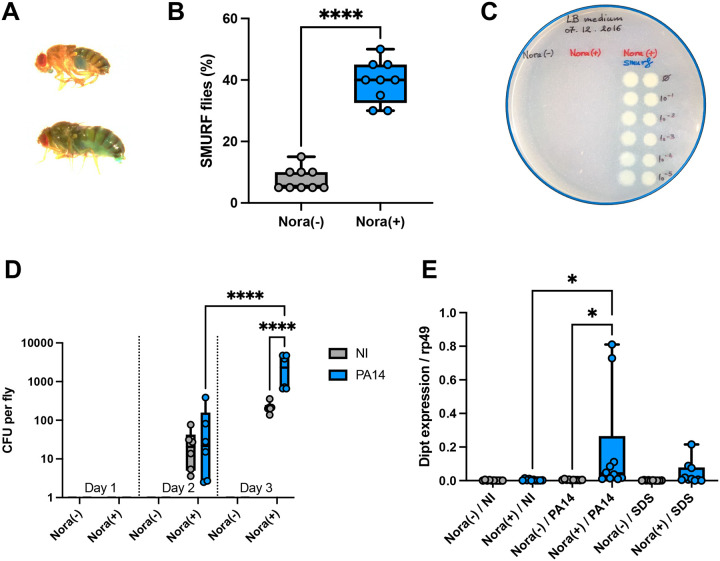
Flies infested with Nora virus show a defect in intestinal barrier integrity (A) Comparison for SMURF assay of 30d old non infected fly (top) and Nora infected fly (bottom). (B) Percentage of SMURF positive flies at 30d on rich food (= LT50 on the survival test in Fig. 2E) (C) Picture of LB plate with drop of hemolymph in dilution series from non infected flies, infected flies with SMURF negative or SMURF positive phenotype. (D) A significant difference of the PA14 titer in the hemolymph was measured at day three of the infection between Nora(−) and Nora(+) flies. (E) A marked increase of *Diptericin* expression in midguts of Nora(+) flies was detected by qRT-PCR after a PA14 intestinal infection at two days or when fed on SDS 1% for 4–6 hours. All data represent the mean of biological triplicates ± SEM of one representative experiment out of three. Mann-Whitney nonparametric t-test was performed for percentage of SMURF flies. One-Way ANOVA with multiple comparison was perform for CFU and Dipt expression quantification. *p<0.05, **p<0.01, ***p<0.001, ****p < 0.0001.

As an increased rate of ISC division may mirror a phenomenon of compensatory proliferation driven by damage to enterocytes, we assessed the permeability of the gut of Nora-positive flies, first using the SMURF assay on 30-day old flies. The percentage of SMURF-positive flies was much higher in Nora-positive flies (Fig. 4A–B). In keeping with this result, when we plated hemolymph collected from flies, we observed a strong microbial growth for that retrieved from SMURF-positive flies, but not from SMURF-negative flies, whether Nora-positive or Nora-negative (Fig. 4C). These results were further confirmed after *P. aeruginosa* ingestion: already after three days, the passage of PA14 through the intestinal epithelium was much higher in Nora-positive than in Nora-negative flies (Fig. 4D). Indeed, there was a detectable induction of a local immune response in the midgut induced by PA14 only in Nora-positive flies at two days instead of around five days for Nora-negative flies (Limmer et al., 2011a), as monitored by measuring *Diptericin* expression levels (Fig. 4E). This immune response is triggered by the proliferation of *P. aeruginosa* in the hemolymph (Chen et al., 2024; Limmer et al., 2011b).

We conclude that the Nora virus affects the integrity of the intestinal epithelium and its barrier function.

### Nora does not directly influence the survival of flies to septic injury

The experiments above show that the presence of the Nora virus in a stock increases its susceptibility to ingested *P. aeruginosa*. Since *P. aeruginosa* ultimately causes a systemic infection, we also tested whether Nora-positive flies would be more sensitive to other types of systemic infections. As shown in Fig. S4A, we did not observe any difference in the survival curves between Nora-positive or -negative flies exposed to spores of the entomopathogenic fungus *Beauveria bassiana*, an infection model in which no macroscopic wounds are inflicted. In contrast, Nora-positive flies succumbed significantly earlier than Nora-negative control flies when challenged with the Gram-positive *Enterococcus faecalis* or the Gram-negative *Enterobacter cloacae* bacterial pathogens in a septic injury model (Fig. S2B–C). However, we found in control experiments that Nora-positive flies pricked with a needle dipped into a sterile PBS solution succumbed at a rate that was similar to that observed in flies submitted to a septic injury, whereas control Nora-negative flies were more resistant to aseptic injury (Fig. S4D). Unexpectedly, *Myd88* flies displayed an enhanced apparent sensitivity to a clean injury, a phenomenon we have observed before and that depends on the endogenous microbiota of *MyD88* flies (Xu et al., 2023). To understand why Nora-infected flies were more susceptible to a near-sterile wound, we measured the proliferation rate of ISCs as it has been reported that injury triggers a ROS-response, in the hypodermis, hemocytes, and in the gut that leads to enterocyte apoptosis and an increased compensatory proliferation of ISCs (Chakrabarti and Visweswariah, 2020; Takeishi et al., 2013). As reported, we observed a modest, statistically not significant, increase of ISC proliferation in Nora-negative flies after injury of the cuticle (Fig. S4E). Nora-positive flies displayed a higher basal rate of ISC proliferation that was however not altered by injury, suggesting that gut damage is unlikely to account for the enhanced sensitivity of Nora-positive flies to wounding of the cuticle. We finally compared the survival rates of flies pricked with a clean needle to that of unchallenged controls and did not find any significant difference (Fig. S4F). We conclude that the apparent sensitivity of Nora-positive flies to clean injuries is actually due to the shortened lifespan of Nora-positive flies. This effect only becomes apparent in systemic infections with mild pathogens that kill the flies slowly (Fig. S4B–C).

### Nora virus is detected in intestinal stem cells in unchallenged flies and adopts an enterocyte localization upon challenge

**Figure 5:**
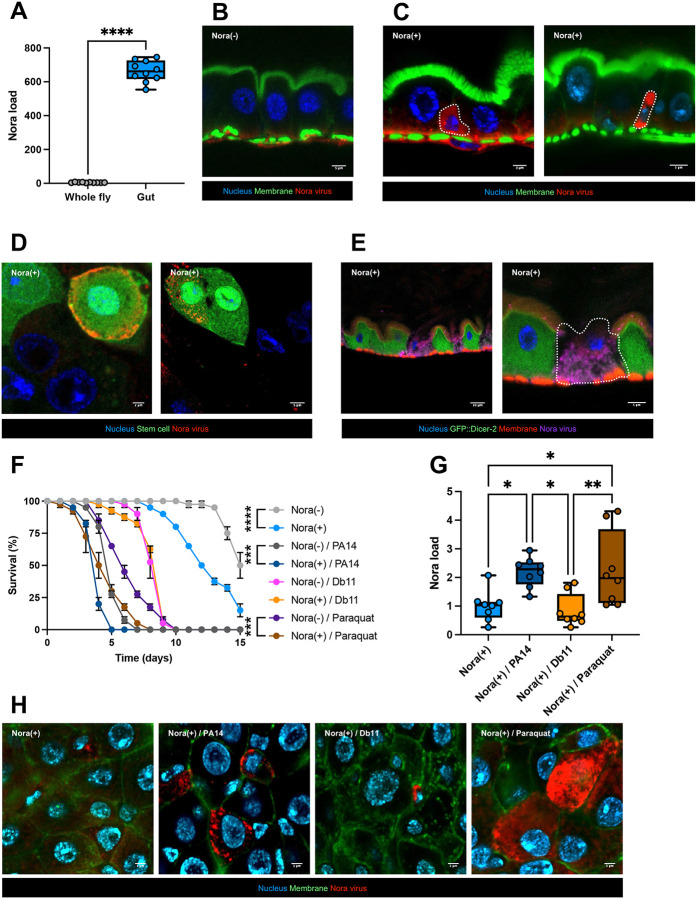
The Nora virus is located in the intestinal stem cells (ISCc) (A) Quantification of Nora virus by RT-qPCR in the whole fly or the intestine only. (B) Confocal pictures of non-infested 5d old intestine. Intestine fixed and stained for DNA (DAPI, in blue), actin (FITC, in green) and Nora (Cy3, in red). The Cy3 antibody (goat anti-mouse) have a background signal in the intestinal muscles. Scale bar = 5 μm. (C) Confocal pictures of infested 5d old intestine. Intestine fixed and stained for DNA (DAPI, in blue), actin (FITC, in green), and Nora (Cy3, in red). Scale bar = 3 μm (left) and 5 μm (right). (D) Colocalization of Nora virus in intestinal stem cells (ISCs) using the reporter *esg-Gal4Gal80*^*ts*^ crossed with *UAS::GFP*. Intestine fixed and stained for DNA (DAPI, in blue), and Nora (Cy3, in red). Scale bar = 2 μm (left) and 5 μm (right). (E) Confocal picture of infected *GFP::Dicer-2* intestine. Intestine fixed and stained for DNA (DAPI, in blue), actin (RFP, in red), Dicer (GFP, in green), and Nora (Cy5, in purple). Scale bar = 10 μm (left) and 5 μm (right). (F) Survival test of flies infected or not by Nora with PA14 or Db11 co-infection or exposure to paraquat. As previously shown, Db11 co-infection with Nora virus has no survival effect. In opposition to PA14 and paraquat where Nora(+) flies are more sensitive. (G) Quantification of Nora virus by RT-qPCR from (F). (H) Confocal picture of Nora infested intestine (Nora(+)) with PA14 co-infection (Nora(+) / PA14), Db11 co-infection (Nora(+) / Db11), and paraquat exposure (Nora(+) / Paraquat). More Nora virus is detected in ECs when combined with PA14 infection or paraquat exposure as shown in (G). Intestine fixed and stained for DNA (DAPI, in blue), actin (FITC, in green) and Nora (Cy3, in red). Scale bar = 3 μm. All data represent the mean of biological triplicates ± SEM of one representative experiment out of three. LogRank test was performed for survival test. Mann-Whitney nonparametric t-test was performed for Nora load. One-Way ANOVA with multiple comparison was perform for Nora load plus pathogen co-infection or paraquat exposure. *p<0.05, **p<0.01, ***p<0.001, ****p < 0.0001.

Since the Nora virus is mostly detected in the gut by RT-qPCR (Habayeb et al., 2009a) and plating assays (Ekstrom and Hultmark, 2016) (Fig. 5A) and since it affects the barrier function of the intestinal epithelium, we raised an antibody against the virus, which is essentially specific except for a cross reaction of the secondary antibodies with intestinal muscles (compare Figs. 5B & S5A to 5C), which precludes drawing any conclusion as regards a possible localization of the Nora virus also in intestinal muscles, as has been previously described for DCV oral infection (Ferreira et al., 2014). In contrast, a positive signal was detected in basally located small triangular cells of the intestinal epithelium of flies that had ingested the Nora virus five days earlier and not in non-infected controls (Figs. 5C, S5A, and 5D). The right panel of Fig. 5C displays a dividing stem cell that yields a basally located ISC and a differentiating progenitor cell (enteroblast or enteroendocrine cell). Indeed, a similar non-basally located small cell is shown in Fig. S5B. We confirmed the localization of the Nora virus to progenitor cells of the intestinal epithelium by staining the Nora virus in *esg- Gal4Gal80*^*ts*^ >*UAS-GFP* flies (Fig. 5D). The Nora-positive signal was always found in the GFP-expressing progenitor cells. Using transgenic fly lines that express Dicer2-fluorescent protein fusions (Donelick et al., 2020; Girardi et al., 2015), we noted that even though the construct was expressed under the direct control of the polyubiquitin promoter, we failed to detect the fusion protein in ISCs (Figs. 5E & S5C), even though GFP is known to be stable In progenitor cells (Fig. 5D). If Dicer-2 were to be unstable specifically in ISCs, this might account for the initial localization of the Nora virus to ISCs, a proposition that would require further experimental confirmation.

We have shown above that Nora-positive flies are more susceptible to *P. aeruginosa* ingestion. We found that the Nora-positive flies were also more susceptible to the strongly oxidizing agent paraquat in survival experiment (Fig. 5F). As for *P. aeruginosa* oral infection, the ingestion of paraquat led to similarly enhanced levels of the viral titer as monitored by RTqPCR (Fig. 5G) and by staining with the Nora antibody (Figs. 5H and S5D). Immunohistochemistry further revealed that the virus starts also proliferating within enterocytes under pathogenic or chemical stress (Fig. 5H). Interestingly, aged flies or young flies raised on rich food also exhibited a growth of the virus in enterocytes (Fig. S5 E–H), in keeping with the increased viral burden found in these 30-day-old flies (Fig. 3A–B). In addition, the detection of the Nora virus in enterocytes also correlated with an enhanced proliferation of ISCs (Figs. 3C & S3D).

In conclusion, the Nora virus is initially restricted to progenitor cells in basal conditions but appears to start proliferating also inside enterocytes upon stress and/or increased proliferation of ISCs. These observations open the possibility that Nora virus become transmitted to differentiating cells after ISC division (Figs. 5C & S5B) and ultimately affects enterocytes.

### ISC division promotes the proliferation of the Nora virus

The above conclusion implies that ISC proliferation is likely needed to promote the proliferation of the virus. To test this hypothesis, we used two complementary approaches that rely on the fact that enterocyte damage triggers the compensatory proliferation of ISCs.

**Figure 6:**
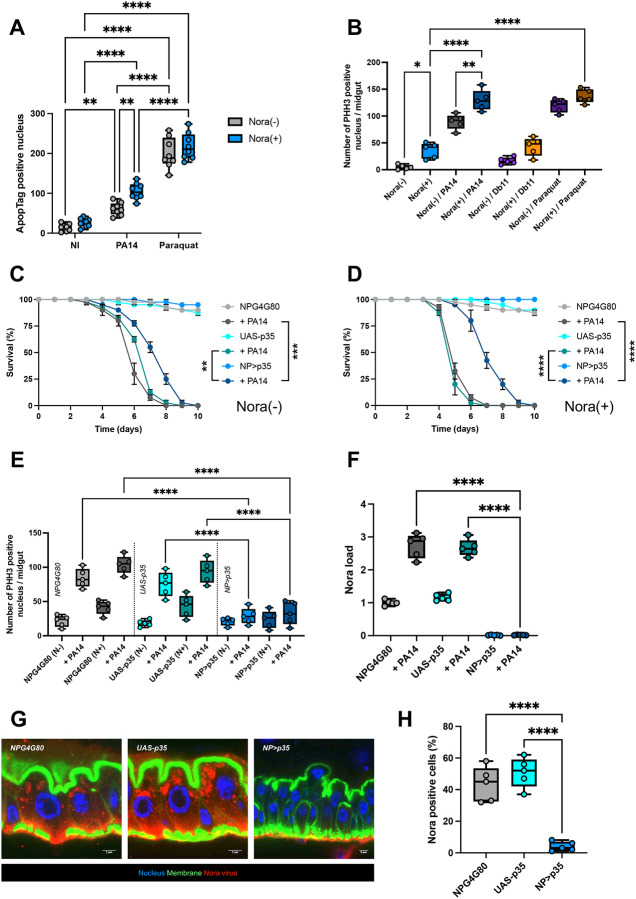
Apoptosis inhibition via p35 expression do not allow Nora virus to spread through the intestine (A) Increased quantification of ApopTag positive nucleus in posterior midgut with PA14 co-infection or paraquat exposure. (B) A significant increase of PHH3 positive nucleus in posterior midgut of Nora infested flies co-infected with PA14. There is a correlation with Nora load increase (Fig. 5G) and PHH3 positive nucleus increase. (C) Survival test of non infested flies with PA14 co-infection in controls (*NP* driver (*NPGal4G80*^*ts*^) and *UAS-p35* alone) and apoptosis inhibition (*NP*>*p35*). (D) Survival test of infested flies with PA14 co-infection in controls (*NP* driver (*NPGal4G80*^*ts*^) and UAS-p35 alone) and apoptosis inhibition (*NP*>*p35*). Inhibition of apoptosis in reducing the lethality induce by co-infection of Nora plus PA14. (E) Significant reduction of PHH3 positive nucleus in posterior midgut from (C) and (D) at 5d post-infection in flies expressing the apoptosis inhibitor p35 in intestine. (F) Quantification of Nora virus by RT-qPCR from (E) at 3d post-infection with low level of Nora virus detected in intestines deficient for apoptotic activity. (G) Confocal picture of infested intestine in control *NP* driver (*NPG4G80*) and *UAS-p35* (*UAS-p35*), or without apoptosis (*NP*>*p35*). The loss of intestinal apoptosis in induce this anarchic structure with enterocytes layers. Intestine fixed and stained for DNA (DAPI, in blue), actin (FITC, in green) and Nora (Cy3, in red). Scale bar = 5 μm. (H) Quantification of Nora positive cells in the intestine. All data represent the mean of biological triplicates ± SEM of one representative experiment out of three. LogRank test was performed for survival test. One-Way ANOVA with multiple comparison was perform for ApopTag, PHH3, and Nora load quantification. *p<0.05, **p<0.01, ***p<0.001, ****p < 0.0001.

First, we measured the degree of apoptosis in epithelial cells through ApopTag/TUNEL staining. We noted that Nora-positive bacteria do not display enhanced levels of apoptosis in this tissue under basal conditions (Fig. 6A, Fig. S6A). However, the ingestion of *P. aeruginosa* led to enhanced levels of apoptosis, significantly more so in Nora-positive flies (Fig. 6A, Fig. S6B). In contrast, paraquat induced much higher levels of positive signals, independently of the Nora status of the exposed flies (Fig. 6A, Fig. S6C). This situation was roughly mirrored in the number of mitotic ISCs (Fig. 6B), although the degree of proliferation induced by paraquat was similar to that induced by *P. aeruginosa* infection, possibly mirroring an adverse effect of paraquat exposure to the division of ISCs (Chatterjee and Ip, 2009).

Next, we attempted to block the induction of apoptosis in enterocytes by expressing there the baculovirus p35 protein that inhibits executioner caspases (Hay et al., 1994). p35 did partially protect the flies from the noxious effects of *P. aeruginosa* ingestion, with a relatively stronger effect on Nora-positive flies, which correlates with the higher number of apoptotic cells measured in those flies (Fig. 6C–D, Fig. 6A). As expected, the expression of p35 in enterocytes blocked the compensatory proliferation of ISCs (Fig. 6E). Strikingly, the Nora virus titer was abolished in both control or *P. aeruginosa*-infected, originally Nora-positive, flies, as monitored by RTqPCR (Fig. 6F) and by immunohistochemistry (Fig. 6G–H) for which less than ten Nora-positive progenitor cells per midgut were detected at best.

**Figure 7:**
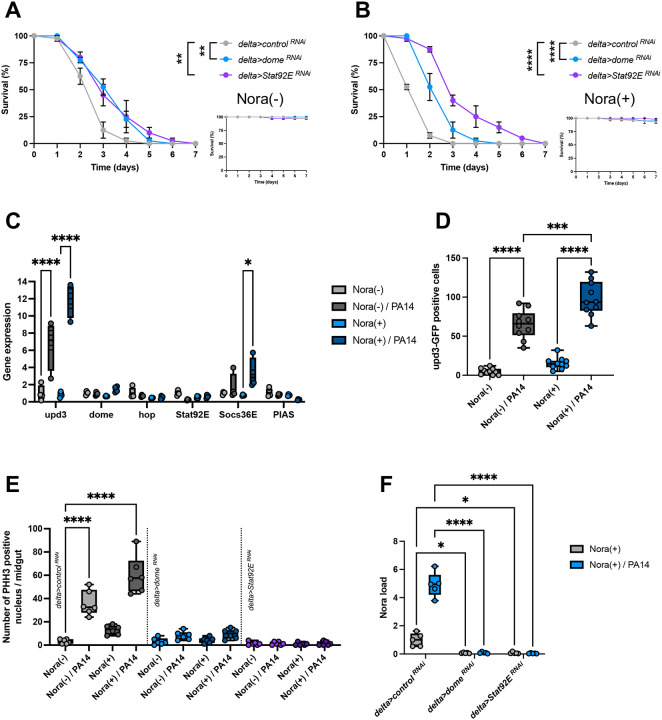
JAK-STAT pathway inhibition does not allow Nora virus to spread through the intestine (A) Survival test of flies non-infested plus PA14 infection with RNA inhibition of JAK-STAT pathway genes showing small survival resistance with *dome* and *Stat92E* RNAi inhibition in the intestinal stem cells using *delta* driver. Non infected control survival test on the right bottom. (B) Survival test of flies infested plus PA14 infection with RNA inhibition of JAK-STAT pathway genes showing significant survival resistance with *dome* and *Stat92E* RNAi inhibition in the intestinal stem cells using *delta* driver. Non infected control survival test on the right bottom. (C) Expression of JAK-STAT genes by RT-qPCR from flies infested or not with PA14 co-infection at 3d post-infection. The *upd3* cytokine is more expressed under PA14 infection. (D) Quantification of *upd3::GFP* in the posterior midgut of flies infested or not with PA14 co-infection at 2d post-infection. As observe in (C), *upd3::GFP* is more detected in presence of PA14 co-infection. (E) Significant loss of PHH3 positive nucleus in posterior midgut at 3d post-infection when the JAK-STAT pathway is altered bu RNAi inhibition of *dome* and *Stat92E* in intestinal stem cells using *delta* driver. (F) Quantification of Nora virus by RT-qPCR from (E) with significant loss of Nora proliferation in intestines with JAK-STAT pathway inhibition in their stem cells. All data represent the mean of biological triplicates ± SEM of one representative experiment out of three. LogRank test was performed for survival test. One-Way ANOVA with multiple comparison was perform for PHH3, gene expression, upd3::GFP, and Nora load quantification. *p<0.05, **p<0.01, ***p<0.001, ****p < 0.0001.

The second approach was to interfere with the signals that drive ISC proliferation upon enterocyte damage. JAK-STAT pathway activation in ISCs promotes their division (Buchon et al., 2009; Cronin et al., 2009; Jiang et al., 2009). Silencing in ISCs the genes that encode either the JAK-STAT pathway receptor Dome or the STAT92E transcription factor also provided protection against ingested *P. aeruginosa*, in both Nora-positive and Nora-negative cells (Fig. 7A–B). Indeed, the *upd3* gene encoding one Dome ligand and the gene encoding the JAK-STAT-regulated inhibitor SOCS36E were induced by *P. aeruginosa* ingestion, again more strongly so in Nora-positive flies (Fig. 7C). This result was further corroborated using a transgenic fly line that expresses an UPD3-GFP reporter protein (Fig. 7D). As expected, silencing *Dome* or *STAT* abolished the compensatory proliferation of ISCs. Again, as for the ectopic expression of p35 in enterocytes, blocking JAK-STAT pathway signaling in ISCs prevented any proliferation of the Nora virus in midguts, whether infected orally with *P. aeruginosa* or not.

## Discussion

**Figure 8:**
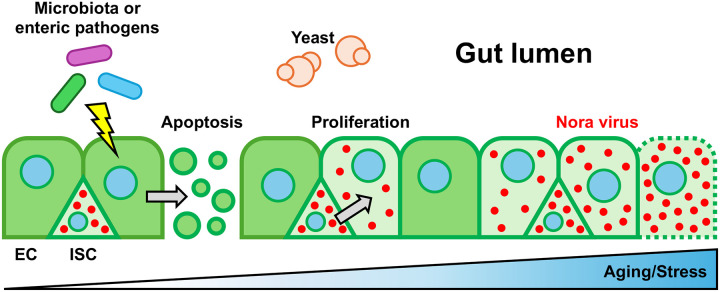
Model of Nora virus propagation in *Drosophila* intestine Schematic representation of Nora virus propagation in the posterior midgut from intestinal stem cells to enterocytes. The Nora virus intially invades ISC in the midgut and remains rather quiescent. External factors such as age, infections or exposure to xenobiotics stress the host intestinal epithelium and induce ISC compensatory proliferation that ensures a degree of homeostasis by replacing damaged enterocytes. ISC cell division appears to stimulate the proliferation of the Nora virus that ultimately contaminates enterocytes. This is likely to further damage the epithelium and lead to a loss of the integrity of this intestinal barrier.

Nora virus infection is prevalent both in wild *D. melanogaster* flies and laboratory stocks (Habayeb et al., 2006; Webster et al., 2015). It has originally been reported to have limited effects on its host fitness, likely due to its proliferation that is mostly restricted to the intestinal epithelium (Habayeb et al., 2009b). Here, in keeping with some more recent work, we report that the presence of Nora virus may be a confounding factor when performing intestinal infections or exposing flies to xenobiotics, and more generally when investigating fitness in extended processes such as aging. Our data support a model according to which Nora virus primarily infects intestinal epithelium progenitor cells and largely remains latent/quiescent. However, exposure to a variety of stresses that all lead to an increased proliferation of ISCs, apparently reactivates the virus and results in the contamination of enterocytes. Ultimately, the generalized proliferation of Nora within epithelial cells affects the barrier function of the midgut epithelium and leads to a decreased fitness that leads to a premature demise of the infected flies (Fig. 8).

Our data document a low basal level of infection in young flies, which correlates with a localization restricted to progenitor cells of the intestinal epithelium. Strikingly, a series of parameters such as age, food nutritional value, pathogenic infection of the gut, and a strong oxidizing agent all induce both an increased viral burden associated to the infection of enterocytes, and proliferation of ISCs. Nora virus appears either to have relatively moderate (Habayeb et al., 2009a; Hanson and Lemaitre, 2023; Nigg et al., 2024; Rogers et al., 2020; Schissel et al., 2021) or intermediate to strong (this study; (Hanson and Lemaitre, 2023)) effects on host fitness. The finding that the higher nutrient quality of the food leads to reduced effects on host fitness may partially account for these differences (Fig. 3D–E), even though the Nora load was much higher on nutrient-rich food (Fig. 3A). The genetic background may also contribute to these differences, even though we observed similar effects of Nora contamination on host fitness in a variety of strains fed on sucrose solution (Fig. S1 F–I). It could also be that the strains used in other laboratories may have modifier genes in their genetic background as one of Oregon strains appeared to be nonpermissive for Nora (Fig. S1J; see also (Hanson and Lemaitre, 2023)). Thus, the Nora load may differ in the different laboratories and could account for the differences in host fitness (Hanson and Lemaitre, 2023). Interestingly, the microbiota had opposite effects on host fitness depending on the food source (positive on sucrose (Fig. S3G) *vs*. negative on standard or rich food (Fig. 3D–E)). As reported earlier, we did observe a higher microbiota titer in Nora-infected flies (Fig. 3C, S3F), which however did not correlate with increased proliferation of ISCs at least on sucrose (Fig. S3H). Of note, strains kept in our laboratory do not contain *Acetobacter* but mostly *Lactobacilli* strains in their microbiota. Whether there is an increased diversity of the microbiota remains to be addressed. An earlier study reported that it was the case in wild-type flies of the *Rel*^*E23*^ strain (Schissel et al., 2021), which however displays a very short life span with some 50% of uninfected flies dead by day 8, possibly due to the presence of the *ebony* marker in this strain.

Our results thus suggest that the virus is activated in dividing progenitor cells, especially when the intestinal epithelium is stressed either by infection or exposure to xenobiotics, and goes on proliferating in differentiated cells derived from these ISCs. The rate of ISC proliferation is always higher in Nora-positive flies, suggesting that Nora contamination of enterocytes may contribute in addition to stressors to their apoptosis and consequently lead to an enhanced compensatory proliferation of ISCs. The finding that blocking apoptosis enhances the fitness of Nora-positive flies to *P. aeruginosa* infection to the level of Nora-negative flies while equally decreasing the proliferation of ISCs supports this proposition. Hence, whenever ISC proliferation is triggered, the additional death of enterocytes caused by Nora virus amplifies the phenomenon in a positive feedback loop. This model is further strengthened by the finding that the JAK-STAT ligand UPD3 is expressed at a higher basal level and induced more strongly by *P. aeruginosa* infection in Nora-positive flies than in Nora-negative flies. Thus, blocking either enterocyte apoptosis or the regulatory pathway that drives compensatory ISC proliferation yields a similar protection against the effects of Nora infection on fly fitness. More importantly, both experimental manipulations lead to a spectacular decrease of the viral titer, even under *P. aeruginosa* infection conditions.

Our experiments point to an important role of initial proliferation of Nora virus in ISCs. With the exception of hematopoietic stem cells that can be infected by retroviruses and herpes viruses, mammalian germ cells, embryonic and adult stem cells have long been known to be rather resistant to viral infections (Wu et al., 2019). Interestingly, stem cells do not appear to rely primarily on interferon-based defenses, even though they do express a subset of interferon-stimulated genes (ISGs) that contribute to the defense against viral infections (Wu et al., 2018). Yet, they are not responsive to interferon, which is mediated through JAK-STAT signaling, and instead rely to a large extent on RNA interference (Maillard et al., 2013; Maillard et al., 2016; Poirier et al., 2021). Whereas interferon signaling in mammals may have adverse effects on stem cell function because of antiproliferative actions of some ISGs, JAK-STAT signaling in *Drosophila* ISCs promotes stem cell division and is also involved in the subsequent differentiation of progenitor cells (Jiang et al., 2009). Interestingly, picornaviruses and coxsackieviruses are known to productively infect activated or proliferating cells while the infection of quiescent cells leads only to persistence or latency (Feuer and Whitton, 2008). Future studies will tell at which step of the cell cycle the Nora virus preferentially proliferates.

We have not addressed here whether the Nora virus is susceptible to host intestinal antiviral defenses. A major antiviral defense in *Drosophila* is the RNAi pathway (Galiana-Arnoux et al., 2006; van Rij et al., 2006), which however appears less active in the gut (Mondotte et al., 2018), albeit oral DCV infection led to an increased viral titer in *Ago2* but not *Dcr2* mutant (Segrist et al., 2021). It will be interesting to determine whether the absence of a Dcr2-fluorescent proteins fusions in progenitor cells that we report in this study rules out a role for the RNAi pathway in intestinal host defense against the Nora virus. Of note, it has been reported that Nora virus infection is not altered in flies defective for the RNAi antiviral pathway (Habayeb et al., 2009b), although the same study failed to see an effect of the JAK-STAT pathway, which conflicts with our own results (this study). The PVR/ERK pathway has been documented to play a role in enterocyte host defense against viral infections (Sansone et al., 2015). However, it needs to be primed by the microbiota through the PGRP-LC/IMD pathway and Pvf2 induction in enterocytes. Given the opposite effects of the microbiota on Nora-infected fly fitness depending on the food source, it appears unlikely that the PVR/ERK signaling pathway plays an essential role in host defense against the Nora virus. In addition, the likely absence of *Acetobacter* species in the microbiota of our fly strains on our food likely limits the induction of Pvf2 expression in enterocytes via the IMD pathway since *Lactobacilli* do not induce it (Sansone et al., 2015). The STING/Relish pathway is another important systemic antiviral pathway (Ai et al., 2024; Cai et al., 2020; Goto et al., 2020; Segrist et al., 2024), which is also relevant to that of the intestinal epithelium. However, current data suggest it is mostly active in enterocytes (Nigg et al., 2024; Segrist et al., 2021). Importantly, in the case of the *Drosophila* A virus (DAV), another prevalent intestinal virus, it is the EGFR pathway and not the JAK-STAT pathway acting in progenitor cells that is important for DAV proliferation. In addition, DAV is mostly found in enterocytes and only occasionally in progenitor cells (Nigg et al., 2024). Thus, while this axis of defense may not be essential once stem cells are proliferating, it might protect the enterocytes in the primary fecal contamination.

As emphasized by other investigators, intestinal viruses may be confounding factors of studies on aging and intestinal physiology, especially as regards the proliferation of ISCs (Hanson and Lemaitre, 2023; Nigg et al., 2024). Our results further add intestinal bacterial infections and exposure to toxicants to this list and more generally any study in which the process under investigation involves monitoring the survival of flies for periods of time over eight days. Finally, we have attempted to establish a Nora-free facility and bleached some 100 fly lines that tested Nora-negative on whole fly extracts. However, after a few months many of these lines tested positive for Nora. It is an open possibility that some fly strains cannot be cured (we had to go through several rounds of bleaching to cure from Nora contamination our Ore-R (SM) stock). Alternatively, a more sensitive test for Nora detection should be used, starting on dissected guts and for instance digital PCR (see also (Nigg et al., 2022)).

## Materials & Methods

### Drosophila stock and husbandry

Wild-type Ore-R(SM) Nora(+) and Ore-R(SC) Nora(−) fly stocks were found in our laboratory. Both stocks of wild-type *Oregon-R* flies tested negative for *Wolbachia* infection. Stock used for the septic injury and natural infections are the following: wild-type *w*^*A5001*^ (Thibault et al., 2004), mutant *MyD88c03881* (Tauszig-Delamasure et al., 2002), mutant DD1 *cn bw* (Rutschmann et al., 2000), *key*^*cO2831*^ (Ferrandon D., unpublished), *NP1-Gal4* (Cronin et al., 2009), *esg-Gal4Gal80*^*ts*^ (Micchelli and Perrimon, 2006), *delta-Gal4* (Zeng et al., 2010), VDRC GD control line (#6000 VDRC), *UAS-dome*^*RNAi*^ (#36355 GD VDRC) (Borensztejn et al., 2013), Bloomington control line *UAS-mCherry*^*RNAi*^ (#35787 Bloomington Drosophila Stock Center) (Borensztejn et al., 2013), *UAS-p35* (#5073 Bloomington Drosophila Stock Center) (Hay et al., 1994; Rahmatika et al., 2019), *UAS-Stat92E*^*RNAi*^ (#26899 Bloomington Drosophila Stock Center) (Nagai et al., 2023), and *upd3::GFP* (Zhou et al., 2013).

All stocks have been checked for the presence of known pathogens and symbiont besides Nora virus (Haller et al., 2014; Lestradet et al., 2014; Niehus et al., 2012). Fly stocks were kept at 25°C and nearly 60% humidity, on a standard semi-solid cormeal medium (6.4% (w/v) cornmeal (Moulin des moines, France), 4.8% (w/v) granulated sugar (Erstein, France), 1.2% (w/v) yeast brewer’s dry powder (VWR, Belgium), 0.5% (w/v) agar (Sobigel, France), 0.004% (w/v) 4-hydroxybenzoate sodium salt (Merck, Germany)). For lifespan analysis, 3 × 20 female flies were kept at 25°C with 60% humidity, on standard fly food. Flies were transferred without anesthesia on fresh food every 4 days. For survival test on a sucrose only diet, 3× 20 female flies were fed on 2 mL on a 50 mM sucrose solution and kept at 25°C with humidity.

### Microbial strains, growth conditions and infection

Microbes were grown in these conditions: *Pseudomonas aeruginosa* PA14 in Brain-Heart-Infusion Broth (BHB), overnight, at 37°C (Rahme et al., 1995); *Serratia marcescens* Db11 (Nehme et al., 2007) in Luria Bertoni Broth (LB), overnight, at 37°C; *Enterobacter cloacae* (Lemaitre et al., 1997), in LB, overnight, at 30°C; *Enterococcus faecalis* (TX0016) in BHB overnight, at 37°C; *Beauvaria bassiana* (Lemaitre et al., 1997)on malt agar plates at 25°C. Intestinal infections were performed as described previously with PA14 (Haller et al., 2014) and Db11 (Lee et al., 2016). Infected and control flies were kept at 25°C. For PA14 infection, 3× 20 female flies were used per experiment. For *E. cloacae* septic injury, 50 mL of an overnight culture of bacteria was centrifuged 30 min at 3 000 × g. The supernatant was removed and pellet used to infect the flies. For *E. faecalis* septic injury, overnight culture was diluted to 1/50 in fresh BHB and allowed to growth for 3 more hours at 37°C. The final culture was centrifuged 10 min at 3000 × g and the pellet washed one time with sterile PBS. The bacteria were resuspended to a final OD = 0.5 that was used to infect female flies with a septic injury. 3× 20 females were infected with each bacterium. The septic injury and the PBS clean injury were performed as described (Haller et al., 2014). 20 females were injured. For *B. bassiana* natural infection, flies were anesthetized, deposited and shaken on a sporulating plate containing the fungus. 3 × 20 females were infected. Flies were kept at 29°C with 60% humidity and transferred, without anesthesia, to fresh food every 2–3 days.

### Dechorionation of *Drosophila* eggs

Nora(+) flies were allowed to lay eggs overnight at 25°C in a cage on apple juice agar plate with yeast paste in the center of the plate. Eggs were collected, washed with water, and dechorionated with a 50% bleach solution for 3 min with constant up and down pipetting of the solution. Eggs were abundantly rinsed with water, aligned under the microscope on a piece of agar medium and transferred by capillarity on a coverslip. One drop of mineral oil was applied to cover the eggs, and the coverslip was deposited on a petri dish with normal *Drosophila* food. After 2 days, larvae were transferred to normal food vial (single bleaching) or treated again with bleach solution as previously and then transferred to normal food vial (double bleaching or even triple bleaching if needed). Once flies emerged, they were tested for Nora virus infection. Usually, there was no difference in the results obtained with the single or double bleaching procedures although this observation applies only to short term observations.

### Pure Nora virus suspension preparation

Nora(+) flies (around 5 000 flies) were crashed in ice cold PBS using a potter homogenizer. The homogenate (around 35 mL) was transferred in a 50 mL Falcon tube and centrifuged at 1 500 rpm for 10 min, at 4°C in an Eppendorf 5810R centrifuge to remove wings, legs and other fly debris. The resulting supernatant was sequentially filtrated through 0.8, 0.45 and 0.22 μm filter units and stored at −80°C. The homogenate (around 26 mL) was then layered in two 15 ml Beckmann Ultra-Clear centrifuge tubes over 1.5 mL of a 30 % (wt/wt) sucrose solution (50 mM Hepes pH 7.0, 0.1 % BSA) and centrifuged at 24,000 rpm for 6.5 hours at 11°C using a JS-24.15 rotor (Beckmann). The pellets were resuspended in a total volume of 500 μL Hepes solution (Hepes 50 mM, pH 7.0) and transferred into a 1.5 mL Eppendorf tube. The solution was then centrifuged at 10,000 rpm for 10 min in a 5424 Eppendorf centrifuge to discard insoluble material. The resulting supernatant containing virus particles was layered over a 40–10 % (wt/wt) sucrose gradient (50 mM Hepes, pH 7.0) prepared in a 15 mL Ultra-Clear centrifuge tube (Beckmann) and centrifuged at 11°C for 4h at 24,000 rpm in a JS-24.15 rotor (Beckmann). An opalescent band, that migrated near the middle of the tube, containing virus particles was then collected by puncturing the tube with a 25-gauge needle mounted on a 1 ml syringe. This solution was then layered onto a 30 % (wt/wt) sucrose solution contained in a 15 mL Beckmann Ultra-Clear centrifuge tubes (50 mM Hepes pH 7.0, 0.1 % BSA) and centrifuged at 24,000 rpm for 6.5 hours at 11°C using a JS-24.15 rotor (Beckmann). The pellet was resuspended in 500 μl of Tris solution (Tris-HCl 10 mM, pH 7.5), aliquoted, and stored at −80°C.

### Nora virus electronic microscopic picture

The sample was prepared by plunge-freezing of 2.5 μL of the sample on a holey carbon Quantifoil R 2/2 grid using FEI Vitrobot Mark IV machine. Images were gained on FEI Polara F30 TEM microscope with acceleration voltage 100 KV and underfocus around 2.0 microns.

### Nora virus re-infection with the pure viral preparation

Nora(−) flies were fed 24 hours with a 1/100 dilution of the viral preparation in sucrose 50mM. In practice, 200 μL of the 1/100 dilution (in sucrose 50mM) of the viral suspension were deposited in an Eppendorf cup that was then placed in an empty fly tube. Flies (male and females) were then added in the tube. The flies were allowed to feed on the viral suspension for 24 hours and then transferred to a fly tube containing standard fly food.

### Nora virus re-infection with feces

200 males of Nora(+) flies were allowed to deposit their feces in a food vial for 5 days at 25°C. Nora(+) flies were then replaced by 50 males and 50 females of cured Nora(+) flies. After 5 days, the vial was emptied, and Nora virus infection status was monitored in those flies (generation G0). Once the progeny emerged, 0 to 4 days old flies were transferred to a fresh vial for 4 days and then monitored for Nora virus titer or used for experiments (first generation after re-infection G1). The same procedure was repeated with the second generation after re-infection (G2).

### Immunostainings

Midguts were dissected in PBS and fixed for 30 minutes with 4% paraformaldehyde. Samples were washed three times with PBS-Triton X-100 0.1% (PBT 0.1%) as described (Socha et al., 2023). For actin staining midguts were incubated for 1h30 at room-temperature or overnight at 4°C in 10 μM Fluorescein Isothiocyanate (FITC) (Sigma-Aldrich #P5282) or Texas-Red labeled phalloidin (Invitrogen TM #T7471). Samples were then washed three times with PBT 0.1%. All samples were mounted on diagnostic microscope slides (Thermo Fisher Scientific) with Vectashield plus DAPI (Vector Laboratories). Samples were observed using a LSM780 confocal microscope (Zeiss) or in Axioskop 2 microscope (Zeiss). All images were analyzed with the ImageJ/Fiji software.

### Phospho-histone H3 immunostaining and microscopy

Fly guts were dissected, fixed, stained by immunohistochemistry with an anti-pH3 antibody (Millipore) and mounted following standard procedures (Lee et al., 2016). Midguts were observed using a fluorescent microscope (Axioskop 2, ZEISS) and nuclei positive for pH3 staining were counted manually.

### Microbiota quantification

Female flies were sterilized for 30 seconds in 70% ethanol and midguts were dissected in sterile PBS (10 midguts per sample) and immediately transferred in sterile PBS. Midguts were homogenized with a sterile pestle. Serial dilutions of the suspension were performed prior to plating on specific solid media and incubated at 30°C. *Acetobacteriaceae* plates, *Enterobacteriaceae* plates and MRS plates were performed as described (Guo et al., 2014). For BHB agar plates: 37 g/L BHB (Sigma), 15 g/L agar (Sigma). Media were autoclaved 15 min, at 121°C (prior to use) and stored at 4°C.

### Bacterial titer in the hemolymph

PA14 presence in the hemolymph was assessed as described previously (Haller et al., 2014). Hemolymphs from 10 female flies per sample were used.

### RT-qPCR analysis of gene expression in *Drosophila* midgut

Fly midguts (without crop, hindgut and Malpighian tubules) were dissected (20 per sample) and RNAs were extracted in TRI Reagent^®^-RT as described (Lee et al., 2016). Reverse transcription was performed using iScriptTM (BIO-RAD). Quantitative PCR was performed using iQTM SYBR^®^ Green (BIO-RAD) and a C1000TM Thermal Cycler (BIO-RAD) device. Expression values were calculated using a standard curve (with genomic DNA) and normalized to *rp49* expression level. Results presented the average ± SD of 3 independent experiments (with biological duplicates or triplicates). See primer sequences in Table 1.

### RT-qPCR analysis of Nora virus titer

Whole flies (6 per sample) were frozen at −80°C. RNA extraction with a Nucleospin^®^ RNA kit (Macherey-Nagel), reverse transcription and quantitative PCR were performed as described (Haller et al., 2014). Quantification values were calculated using a standard curve obtained from serial dilutions of a plasmid carrying the Nora virus DNA sequence amplified by the couple of primers used for the PCR. See primer sequences in Table 1.

To check the absence of other potentially contaminating viruses in the purified Nora virus preparations, we used the set of primers described by (Wu et al., 2010).

### Antibiotics treatment

3× 20 female flies (3–8 days old) were kept on a sucrose (50mM) only diet with addition of a 5 antibiotics cocktail: 100 μg/mL ampicillin, 50 μg/mL vancomycin, 100 μg/mL neomycin, 100 μg/mL metronidazole and 50 μg/mL tetracyclin. Tubes were changed every 3 days and flies transferred without anesthesia.

### SMURF

The SMURF assay was developed to observe the integrity of the intestinal barrier (Rera et al., 2011). Flies are kept on a sucrose solution colored with Blue food dye (Brilliant Blue FCF E133). After ingestion, if there is an intestinal integrity loss, the dye will diffuse in the hemolymph, coloring the fly in blue.

### ApopTAG

Used as described by the manufacturer Sigma-Aldrich for the product ApopTag Fluorescein In Situ Apoptosis Detection Kit.

### JAK-STAT gene expression

Fly midguts (without crop, hindgut and Malpighian tubules) were dissected (20 per sample) and RNAs were extracted in TRI Reagent^®^-RT as described (Lee et al., 2016). Reverse transcription was performed using iScriptTM (BIO-RAD). Quantitative PCR was performed using iQTM SYBR^®^ Green (BIO-RAD) and a C1000TM Thermal Cycler (BIO-RAD) device. Expression values were calculated using a standard curve (with genomic DNA) and normalized to *rp49* expression level. Results presented the average ± SD of 3 independent experiments (with biological duplicates or triplicates).

### Statistical analysis and reproducibility

All statistical analyses were performed on Graphpad Prism version 10 (Graphpad software Inc., San Diego, CA). The Mann–Whitney tests were used unless otherwise indicated. For survival experiments, the time it takes for 50% of the flies to succumb (LT50) was determined for each curve being compared. An unpaired *t*-test used on the LT50s from biological triplicates was used to assess the significance between two survival curves. When using parametric tests (analysis of variance (ANOVA) and *t*-test), a Gaussian distribution of data was checked using either D’Agostino-Pearson omnibus, Anderson-Darling, or Shapiro-Wilk normality tests. All experiments were performed at least three times. Significance values: *p < 0.05, **p < 0.01, ***p < 0.001, ****p < 0.0001.

## Supplementary Material

Supplement 1

Supplement 2
