# Supplementary material for: Nora virus proliferates in dividing intestinal stem cells and sensitizes flies to intestinal infection and oxidative stress
Source: bioRxiv. 2025 Feb 4:2025.01.30.635658. Preprint. [Version 2] doi: 10.1101/2025.01.30.635658 (PMC11838516; doi:10.1101/2025.01.30.635658)

Figure Sup 1:

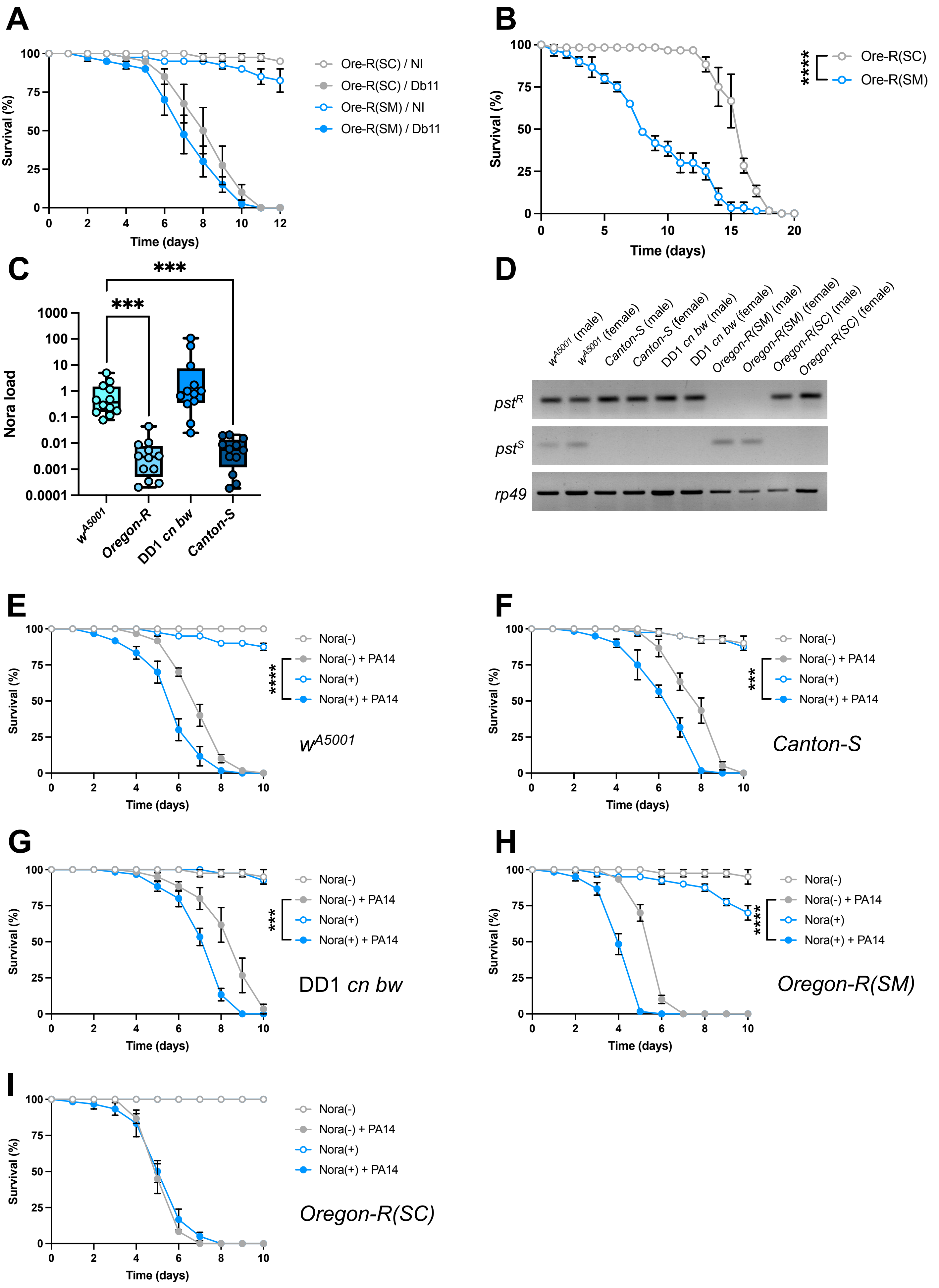

A
Survival (%)
Time (days)
Ore-R(SC) / NI
Ore-R(SC) / Db11
Ore-R(SM) / NI
Ore-R(SM) / Db11
B
Survival (%)
Time (days)
****
Ore-R(SC)
Ore-R(SM)
C
***
***
Nora load
wA5001
Oregon-R
DD1 cn bw
Canton-S
D
wA5001 (male)
wA5001 (female)
Canton-S (male)
Canton-S (female)
DD1 cn bw (male)
DD1 cn bw (female)
Oregon-R(SM) (male)
Oregon-R(SM) (female)
Oregon-R(SC) (male)
Oregon-R(SC) (female)
pstR
pstS
rp49
E
Survival (%)
Time (days)
Nora(-)
Nora(-) + PA14
Nora(+)
Nora(+) + PA14
****
wA5001
F
Survival (%)
Time (days)
Nora(-)
Nora(-) + PA14
Nora(+)
Nora(+) + PA14
***
Canton-S
G
Survival (%)
Time (days)
Nora(-)
Nora(-) + PA14
Nora(+)
Nora(+) + PA14
***
DD1 cn bw
H
Survival (%)
Time (days)
Nora(-)
Nora(-) + PA14
Nora(+)
Nora(+) + PA14
****
Oregon-R(SM)
I
Survival (%)
Time (days)
Nora(-)
Nora(-) + PA14
Nora(+)
Nora(+) + PA14
Oregon-R(SC)

Figure Sup 2:

A

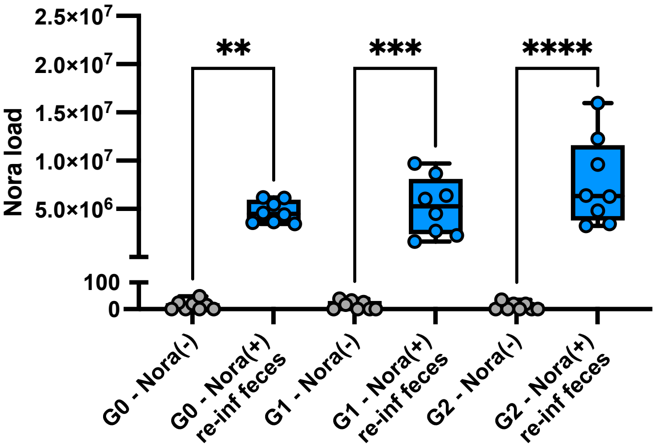

B

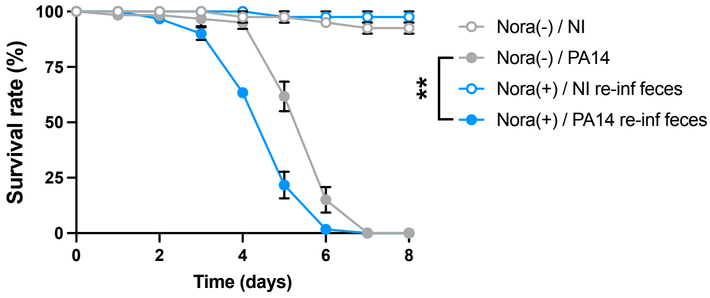

C

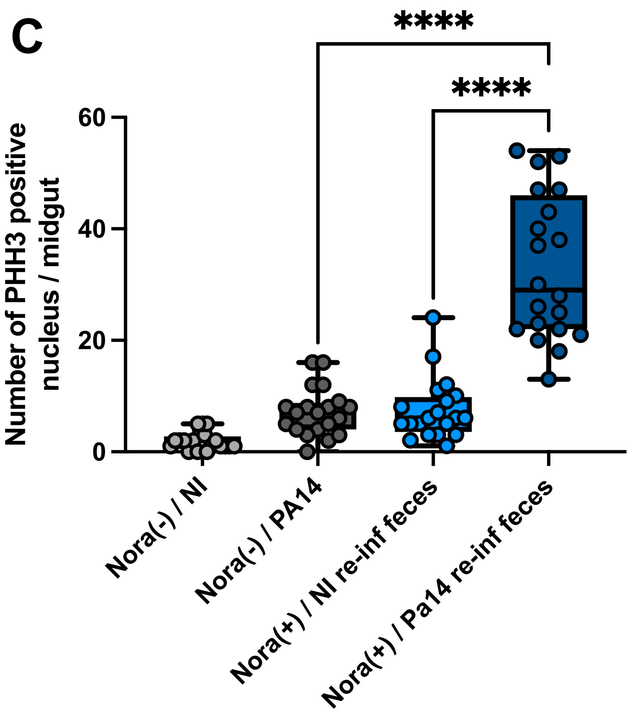

Figure Sup 3:

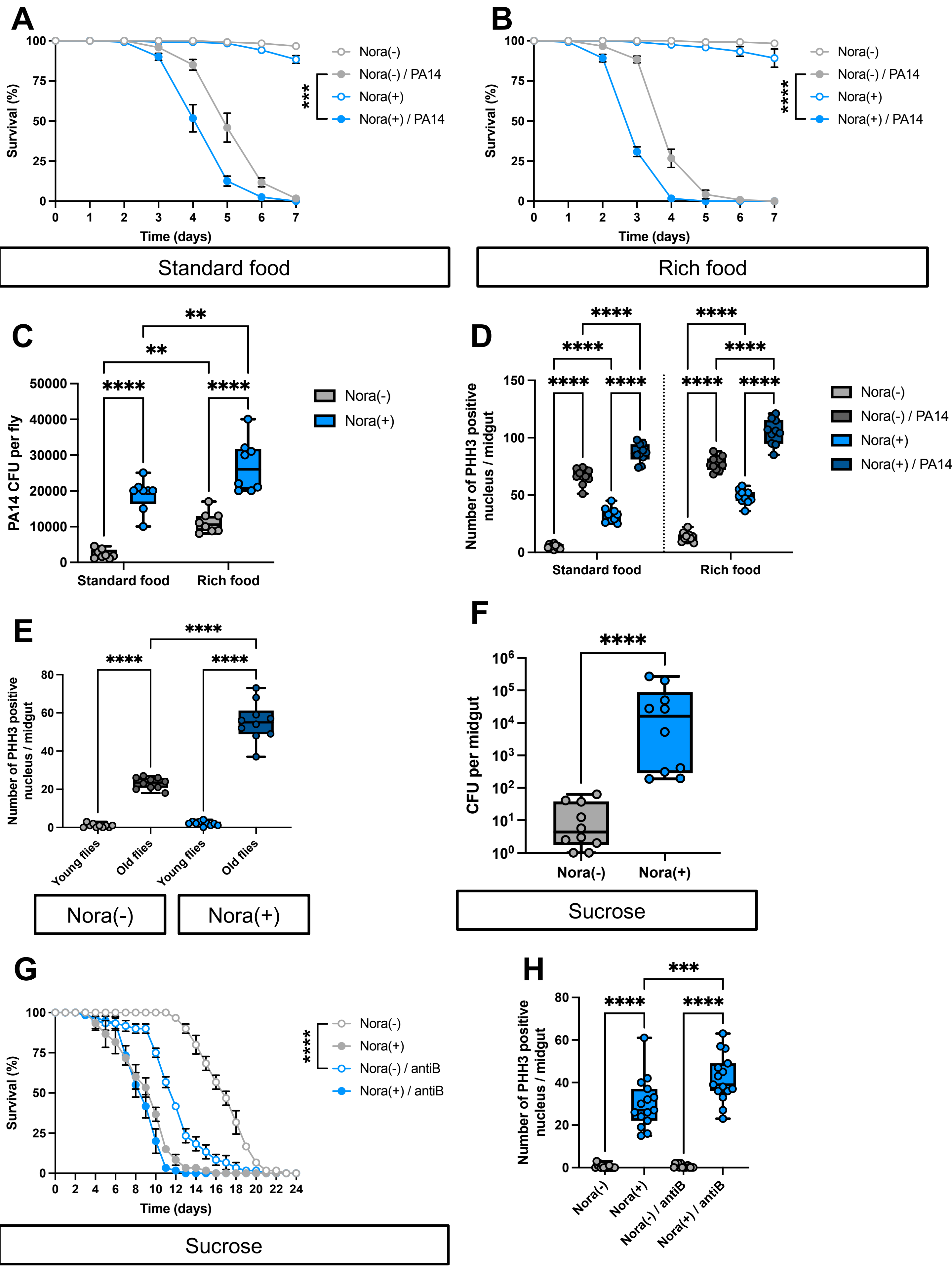

A
Survival (%)
Time (days)
Nora(-)
Nora(-) / PA14
Nora(+)
Nora(+) / PA14
***
Standard food
B
Survival (%)
Time (days)
Nora(-)
Nora(-) / PA14
Nora(+)
Nora(+) / PA14
****
Rich food
C
PA14 CFU per fly
Standard food
Rich food
Nora(-)
Nora(+)
D
Number of PHH3 positive nucleus / midgut
Standard food
Rich food
Nora(-)
Nora(-) / PA14
Nora(+)
Nora(+) / PA14
E
Number of PHH3 positive nucleus / midgut
Young flies
Old flies
Young flies
Old flies
Nora(-)
Nora(+)
F
CFU per midgut
Nora(-)
Nora(+)
Sucrose
G
Survival (%)
Time (days)
Nora(-)
Nora(+)
Nora(-) / antiB
Nora(+) / antiB
****
Sucrose
H
Number of PHH3 positive nucleus / midgut
Nora(-)
Nora(+)
Nora(-) / antiB
Nora(+) / antiB

Figure Sup 4:

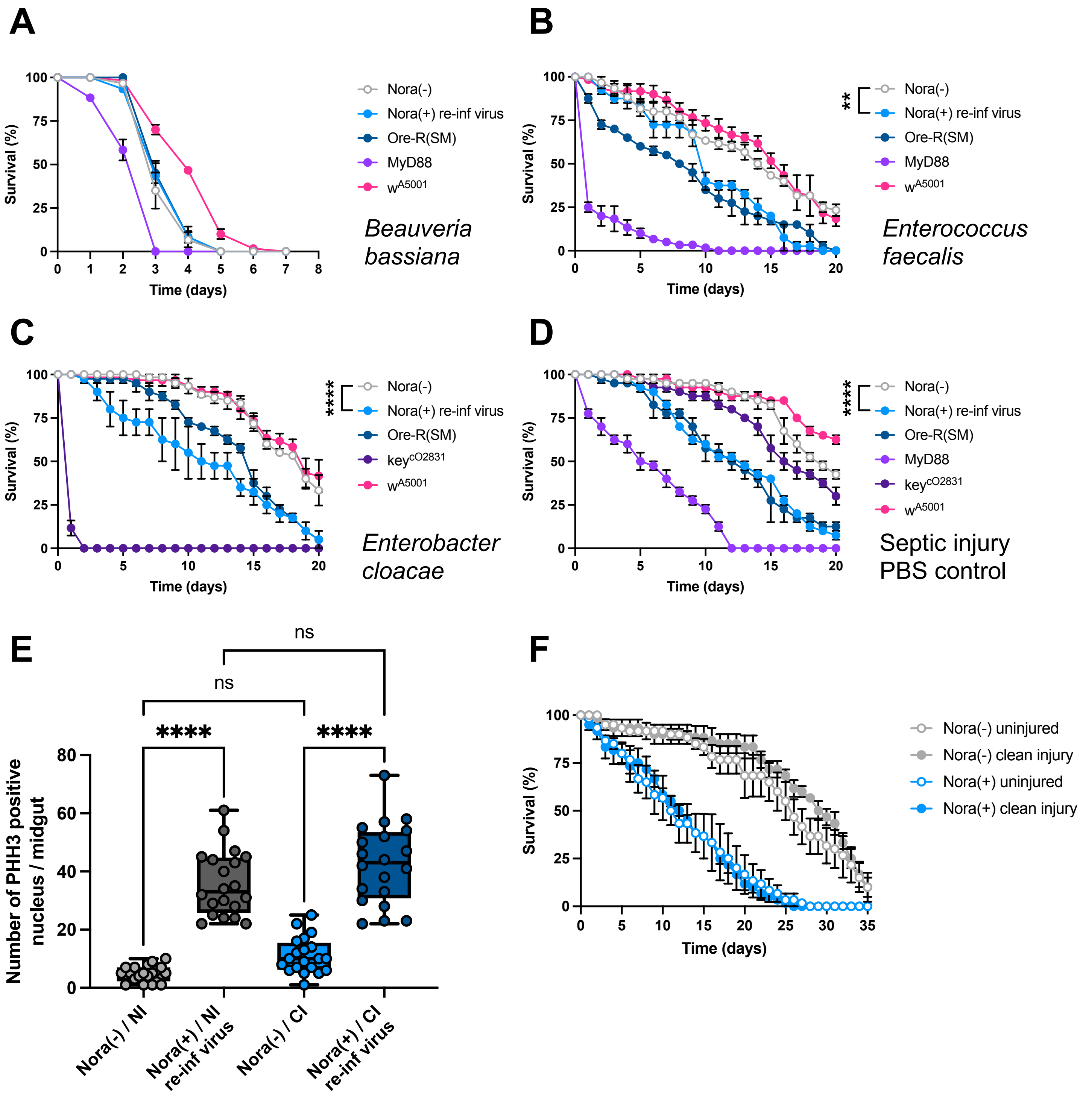

A
B
C
D
E
F
Survival (%)
Time (days)
Nora(-)
Nora(+) re-inf virus
Ore-R(SM)
MyD88
w^A5001
key^cO2831
Beauveria bassiana
Enterococcus faecalis
Enterobacter cloacae
Septic injury PBS control
**
****
Number of PHH3 positive nucleus / midgut
ns
Nora(-) / NI
Nora(+) / NI re-inf virus
Nora(-) / CI
Nora(+) / CI re-inf virus
Nora(-) uninjured
Nora(-) clean injury
Nora(+) uninjured
Nora(+) clean injury

Figure Sup 5:

A

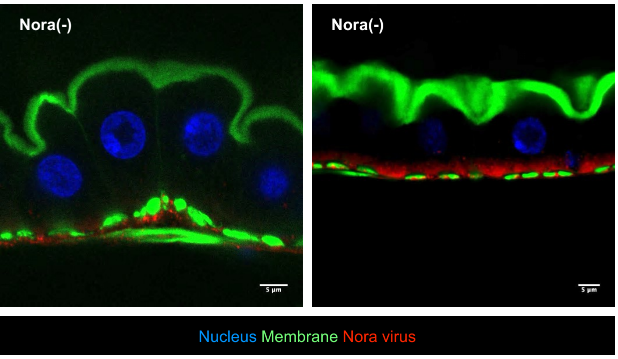

B

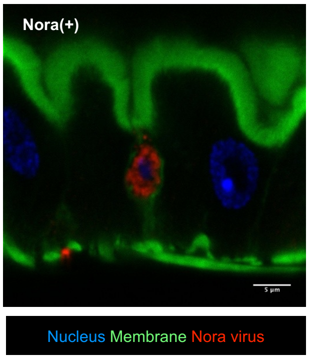

C

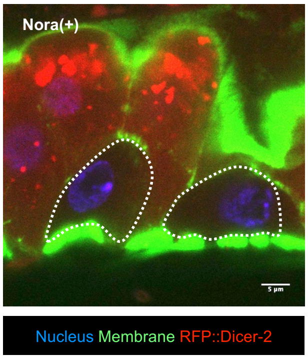

D

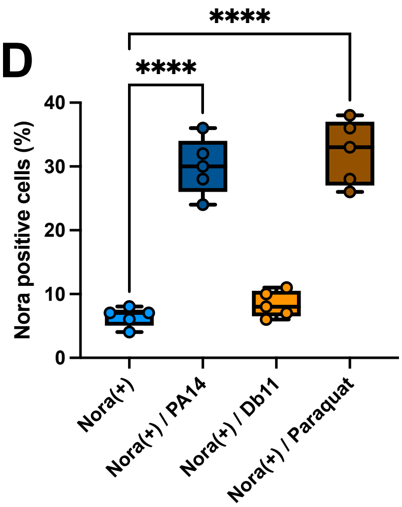

E

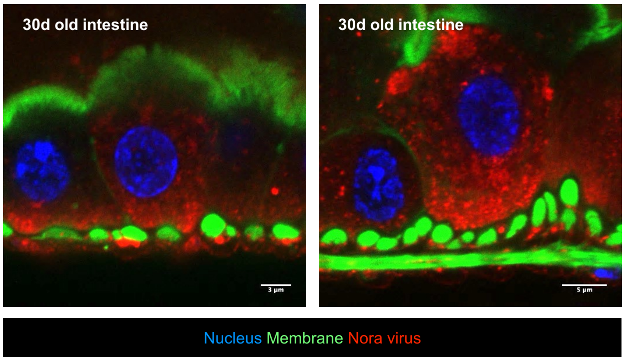

F

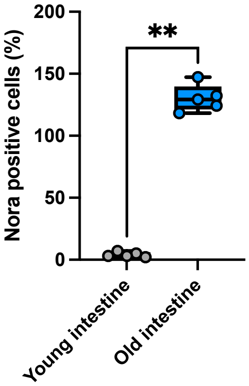

G

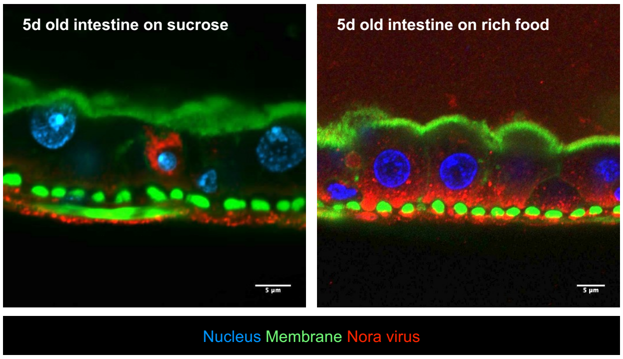

H

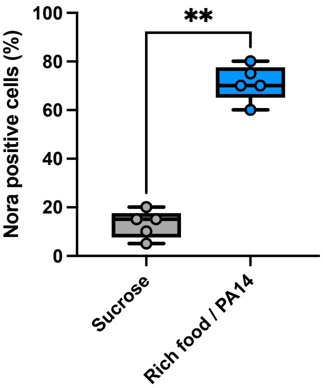

Figure Sup 6:

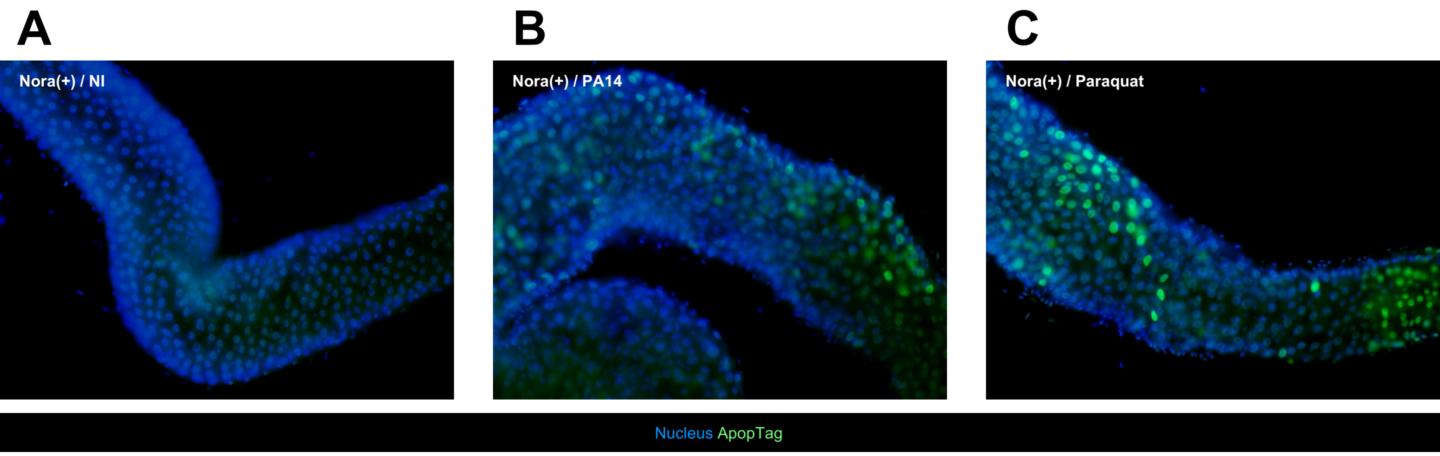

Supplement: Supplement 1 [file media-1.pdf]
