# Supplementary material for: Nora virus proliferates in dividing intestinal stem cells and sensitizes flies to intestinal infection and oxidative stress
Source: bioRxiv. 2025 Feb 4:2025.01.30.635658. Preprint. [Version 2] doi: 10.1101/2025.01.30.635658 (PMC11838516; doi:10.1101/2025.01.30.635658)
Supplement: Supplement 2 [file media-2.pdf]

Name	Sequence (5’-3’)
rp49 Fw (also known as RpL32)	GACGCTTCAAGGGACAGTATCTG
rp49 Rv (also known as RpL32)	AAACGCGGTTCTGCATGAG
Nora Fw	AACCTCGTAGCAATCCTCTCAAG
Nora Rv	TTCTTGTCCGGTGTATCCTGTATC
Diptericin Fw	GCTGCGCAATCGCTTCTACT
Diptericin Rv	TGGTGGAGTGGGCTTCATG
upd3 Fw	CGACCTGCAGATTTACGTGG
upd3 Rv	GGTCCCAGTGCAACTTGATG
dome Fw	TGACCGATACATTCCGCGTA
dome Rv	GTAGGTGATGGGCTTCTCGT
hop Fw	GATGAGACCAAGCGCTTCAG
hop Rv	TTTCCGCCTTGATCGTGTTG
Stat92E Fw	CGTTACGCGCAATACACAGA
Stat92E Rv	CGTTTTGAATCTCGCCCGAT
Socs36E Fw	GACAAATCGAACTGCTGGCA
Socs36E Rv	CGTTGTTATTCACGGGCTGT
PIAS Fw (also known as Su(var)2-10)	ACTGTCTGGCCGTATACCTG
PIAS Rv (also known as Su(var)2-10)	CCCTTCGTCTTCATTCGCTG
DCV Fw	TCATCGGTATGCACATTGCT
DCV Rv	CGCATAACCATGCTCTTCTG
FHV.1 Fw	TTTAGAGCACATGCGTCCAG
FHV.1 Rv	CGCTCACTTTCTTCGGGTTA
FHV.2 Fw	CAACGTCGAACTTGATGCAG
FHV.2 Rv	GCTTTACAGGGCATTTCCAA
VSV Fw	CATGATCCTGCTCTTCGTCA
VSV Rv	TGCAAGCCCGGTATCTTATC
Sinv Fw	CAAATGTGCCACAGATACCG
Sinv Rv	ATACCCTGCCCTTTCAACAA
CrPV-1 Fw	GCTGAAACGTTCAACGCATA
CrPV-1 Rv	CCACTTGCTCCATTTGGTTT
CrPV-2 Fw	GGAATTTTTGGAGACGCAAA
CrPV-2 Rv	GTGAAGGGGGCAACTACAAA
DAV-1 Fw	CGAACTGCCAACTGAGGTCT
DAV-1 Rv	CCACCCCGGTTGTTAATGGA
IIV6 Fw	TTGTTAGGAATTGGAACTGGAA
IIV6 Rv	GCCCTAGATGCTGCTTGTTC
DBV-A Fw	TGCAGTCAGACGCCAGTATC
DBV-A Rv	CCCCTGAACCTGGTAGCATA
DTV Fw	AGTTTTGGGATTGGCAACAG
DTV Rv	TTACGCCCCTTGAATGGTAG
DXV-A Fw	CATCGTCGACATCACCAAAC
DXV-A Rv	TCCTGTGAAAGCTGCAAATG
DXV-B Fw	TGAGCAAAATTACGCACAGG
DXV-B Rv	CCATACGCGTTGTGTATTCG
